# Decrease in Cavity Size and Oligodendrocyte Cell Death Using Neurosphere-Derived Oligodendrocyte-Like Cells in Spinal Cord Contusion Model

**DOI:** 10.22034/ibj.22.4.246

**Published:** 2018-07

**Authors:** Hojjat Allah Abbaszadeh, Taki Tiraihi, Yousef Sadeghi, Ali Reza Delshad, Majid Sadeghizadeh, Taher Taheri, Ali Noori-Zadeh

**Affiliations:** 1 Hearing Disorders Research Center and Department of Biology and Anatomical Sciences, School of medicine, Shahid Beheshti University of Medical Sciences, Tehran, Iran; 2 Department of Anatomical Sciences, Faculty of Medical Sciences, Tarbiat Modares University, Tehran, Iran; 3 Department of Anatomy, Shahed University, Tehran, Iran; 4 Department of Genetics, School of Basic Sciences, Tarbiat Modares University, Tehran, Iran; 5 Shefa Neurosciences Research Center, Khatam Al-Anbia Hospital, Tehran, Iran; 6 Department of Clinical Biochemistry, Faculty of Paramedicine, Ilam University of Medical Sciences, Ilam, Iran

**Keywords:** Myelin basic protein, Oligodendrocyte, Spinal cord injury

## Abstract

**Background:**

Oligodendrocyte cell death is among the important features of spinal cord injury, which appears within 15 min and occurs intensely for 4 h after injury, in the rat spinal contusion model. Accordingly, the number of oligodendrocytes progressively reduced within 24 h after injury. Administration of oligodendrocyte-like cells (OLCs) into the lesion area is one of the approaches to counterbalance this condition.

**Methods:**

Bone marrow stromal cells were transdifferentiated into neurospheres and then into neural stem cells and later were differentiated into OLCs using triiodothyronine and transplanted into the spinal cord contusion rats. The post-injury functional recovery was explored and compared with the control group using Basso-Beattie-Bresnahan and narrow beam behavioral tests. At the end of 12^th^ week, spinal cord segments T12-L1 were histomorphologically studied by immunohistochemistry.

**Results:**

Motor improvement was more obvious during 2^nd^ to 4^th^ weeks and got less prominent during 4^th^ to 12^th^ weeks. Histomorphometric findings indicated that cavity formation decreased in epicenter of transplantation area in experimental groups in comparison with the control groups.

**Conclusion:**

The findings obtained in the present study showed that OLC therapy is a potential approach in the treatment of spinal cord traumatic injuries.

## INTRODUCTION

Spinal cord injury (SCI) is characterized by hemorrhage, ischemia, and edema with subsequent tissue damage due to cellular death[1]. Oligodendrocyte death is an important factor in nerve regeneration failure in SCI occurring immediately after the onset of a traumatic insult[2,3]. The number of oligodendrocyte is reduced within 24 h after injury and persistently declined three to seven days afterwards[4]. It has recently been known that a variety of factors can bring about detrimental effects on oligodendrocytes in acute lesions sites, including blood components, which can trigger either necrosis or apoptosis of the oligodendrocyte progenitor cells dampening their proliferation and migration. Meanwhile, damaged vessels or released proteolytic enzymes from necrotic cells can activate degeneration of neighboring oligodendrocytes. Ischemia and reperfusion are also major culprits of free radicals propagation, such as reactive oxygen and nitrogen species[5-8]. Moreover, excitotoxicity provokes oligodendrocyte death by triggering cell calcium overload and initiation of multiple intracellular pathways through glutamate receptor subtypes, including 2-amino-3-(5-methyl-3-oxo-1, 2-oxazol-4-yl) propanoic acid, N-methyl-D-aspartic acid, and kainate receptors[9,10]. Cytokines such as tumor necrosis factor-α and IL-1β can both exacerbate excitotoxicity and be up-regulated within minutes after SCI by impeding the glutamate re-uptake[11]. Lymphocytes can directly lyse oligodendrocytes and propagate apoptosis by releasing molecules that activate death receptors via interferon gamma, IL-2, and tumor necrosis factor-α[12]. The latter may serve as an external signal enhancing neurons and oligodendrocytes apoptosis after SCI. Death receptors such as Fas and p75 nerve growth factor receptor are activated in the presence of their respective ligands, i.e. FasL and nerve growth factor, respectively. These factors may also cause oligodendrocyte death[13].

Collectively, many factors exert their detrimental effects on oligodendrocyte cell viability, and one of the conceptions in this field is alleviating oligodendrocyte loss during CNS trauma by administration of oligodendrocyte-like cells (OLCs) into the lesion area. In the present study, bone marrow stromal cells (BMSCs) were transdifferentiated into neurospheres and then differentiated into OLCs. The OLCs were transplanted in the contused spinal cord investigating the functional recovery after cell transplantation.

## MATRIALS AND METHODS

### Animals

In total, 51 inbred female Wistar rats with an average weight of 200-250 g were used in this study; 48 rats were used for rat model experimental groups and 3 for cell preparations. During the examinations, rats were maintained under a 12 h/12 h light/dark cycle and were given food and water *ad libitum*. The animal studies were carried out in accordance with the Helsinki Declaration on animal experimentation and were approved by the ethical committee of Shahid Beheshti University of Medical Sciences (Tehran, Iran).

### Oligodendrocyte-like cell generation

As previously described in details[14], the femurs and tibias of sacrificed rats were isolated, and their whole bone marrow was aspirated using a syringe needle (18 G) containing 3-4 ml of DMEM (Stem Cell Technology Company, USA) supplemented with FBS (Gibco, USA) and 0.25% Trypsin/1 mM EDTA (Gibco). The whole bone marrow, including BMSCs, was transferred into 75-cm^2^ plastic flasks (Nunc, Denmark) containing DMEM/F12 (Stem Cell Technology Company) supplemented with 10% FBS, 100 U/ml penicillin (Invitrogen, USA), 100 mg/ml streptomycin (Invitrogen, USA), and 2 mM/ml L-glutamine (Gibco). Following incubation in a humidified incubator using 5% CO_2_ at 37°C for 24 h, the non-adherent cells were discarded. After reaching 90% confluency, BMSCs were harvested using 0.25% Trypsin/1 mM EDTA (Gibco) at 37°C for 5 min to obtain a single cell suspension. The cells were then re-plated (4000 cells/cm^2^) for four passages. Next, the BMSCs were dissociated using 0.25% Trypsin/1 mM EDTA (Gibco, USA) and plated on plastic flasks at a density of 5000 cells/cm^2^ in the neurosphere formation medium, DMEM/F12. The medium was supplemented with 2% B27 (Invitrogen), 20 ng/ml basic fibroblast growth factor (bFGF; Sigma-Aldrich, Germany), 20 ng/ml epidermal growth factor (Sigma-Aldrich, Germany), 100 U/ml penicillin, and 100 mg/ml streptomycin. The neurosphere-like clusters were observed after two days of incubation. For neural stem cell (NSC) generation, the neurospheres were harvested using 0.25% Trypsin/1 mM EDTA seven days after plating and seeded on the poly-L-lysine-coated coverslips (Sigma-Aldrich) in 6-well culture plates. The plates contained DMEM/F12 medium supplemented with 5% FBS, 10 ng/ml epidermal growth factor, 10 ng/ml bFGF, 1% B27, 100 U/ml penicillin, and 100 mg/ml streptomycin. The cells were incubated in the DMEM/F12 induction medium containing the platelet-derived growth factor (PDGF-AA), bFGF, and heregulin (5, 10, and 200 ng/ml, respectively; all from Sigma-Aldrich) for 2 days, resulting in oligodendrocyte progenitor-like cells differentiation. Subsequently, to differentiate oligodendrocyte progenitor-like cells into OLCs, the cells were inducted with triiodothyronine in 5% CO_2_ at 37°C for 2 days.

### Immunocytochemical method

The cultured cells were fixed with 4% paraformaldehyde (Sigma-Aldrich) in 0.1 M phosphate buffer (pH 7.4) for approximately 20 minutes. Permeabilized cells were then blocked with 5% BSA (Sigma-Aldrich) for 30 minutes. Immunostaining were performed on induced cells. We exerted both mouse anti-O4 monoclonal antibodies and mouse anti-oligo2 monoclonal antibody (both 1:100 dilution; Millipore Corporation), the particular marker for immature OLCs and mouse anti-myelin basic protein (MBP) monoclonal antibody (1:1000, Millipore Corporation), a particular marker for mature oligodendrocytes. The cells were incubated with fluorescein isothiocyanate (FITC)-conjugated rabbit anti-mouse secondary antibody (1:100; Millipore Corporation) at room temperature for 2 h. The cells were counterstained by ethidium bromide (Sigma-Aldrich) for one minute.

### Quantitative real-time RT-PCR (qRT-PCR)

Using Ambion kit (Invitrogen, USA), total RNA was extracted from rat neonate brain (control group) and OLCs following the manufacturer’s instructions. Subsequently, each sample of separated RNA was treated with DNase I enzyme (Invitrogen). The spectrophotometry (UV absorbance of 260/280 nm) and denaturing agarose gel electrophoresis were applied to evaluate the quality of extracted total RNA. The Revert Aid First Strand cDNA Synthesis Kit (Fermentas, Lithuania) was used for cDNA generation based on the instructions provided by the manufacturer. In order to assess the possibility of DNA contamination, a control RT-minus reaction containing all components of cDNA synthesis reaction, except for the reverse transcriptase enzyme, was prepared and used. qRT-PCR using SYBR Green PCR Master Mix (Applied Biosystems, USA) detection method using a Step One real-time PCR machine (Applied Biosystems). Glyceraldehyde-3-phosphate dehydro-genase was used as an internal control gene for normalizing the values. The qRT-PCR primers are shown in Table 1.

**Table 1:** Sequence of primers used in the RT-PCR and the qRT-PCR amplifications

Gene	Primer type	Sequence	Annealing temperature	Product size (bp)
*PDGFRα*	F R	CTAATTCACATTCGGAAGGTTG GGA CGATGGGCGACTAGAC	57	175
*MOG*	F R	GAGCTCATTATGGCCTTTCATGG GTTCCTGCGTGAACAGTCCAC	62	180
*OLIG2*	F R	GACGACATTATGGGCTTTGATGG GTTTCTGCCTGAACAGTCCAC	62	170
*GAPDH*	F R	CCACAACTC TTCCATTCTC CCAAGATTCACGGTAGATAC	62	200

### Surgical procedures

Rats were anesthetized using intraperitoneal injection of ketamine and xylazine (80 and 10 mg/kg, respectively; both from Sigma-Aldrich). To expose rat spinal cord, the dorsal laminectomy was performed at the T12-L1 level[15,16]. The contusion model of traumatic spinal cord was induced by dropping a 10-g metal rod with 2 mm diameter from 25-mm height. Eight days post injury, the rats were randomly divided into six groups (eight rats in each group): laminectomy only or sham operated (S), contusion only (C1: C), contusion+ normal saline (C2: CN), contusion+BMSCs trans-plantation (E1:CB), contusion+NSCs trans-plantation (E2:CNSCs), and contusion+neurosphere-derived OLCs transplantation (E3:COLCs).

### Transplanted-cell labeling

At the third passage, when BMSCs and NSCs reached 70% confluence, the culture medium was removed and replaced with a medium containing 10 μM BrdU (Sigma-Aldrich), which was kept for 48 h. The cells from the first passage were BrdU-labeled and collected as they reached 90% confluence and subsequently washed with PBS (Gibco) for further experimentation.

### Surgical procedures and cell transplantations

The cell injections were performed on the eighth day post contusion induction. The rats were re-anesthetized as previously described[1], and the laminectomy sites were re-exposed again. From the cell culture plates, the BrdU-labeled cells (BMSCs and NSCs) were collected as a single-cell suspension using Trypsin and EDTA (Gibco). Using a 30-G needle and a nanoinjector pump (Stoelting Co., Wood Dale, IL, US), each cell injection was performed at the rate of 25 µl/min. Each rat from the CB, CNSCs, and COLCs groups was intraspinally injected with 300,000 cells/9 μl normal saline at the epicenter, rostral, and the caudal regions of the treatment site (each site received 100,000 cells/3 μl). In the CN group, only 9 μl normal saline was injected under the aforementioned conditions. The rats were allowed to survive 11 weeks post transplantation.

### Behavioral assessment

The open-field locomotor testing was performed using Basso-Beattie-Bresnahan (BBB) rating scale for a total of 12 weeks, i.e. from the assigning of the rats into experimental groups, after the SCI induction, and once a week for 11 weeks after cell transplantations. All of the animals were coded, and the behavioral assessments were performed by two investigators blinded to the experimental groups. The mean BBB scores were enumerated by the injured groups and then plotted as a function of time after the SCI induction.

### Sensory-motor test

By conducting narrow beam tests, an extra measurement for recovery assessment was performed as previously described[17]. The rats were trained to traverse a beam (4 cm wide and 80 cm high) in advance for 5 days (5 min per day). The ability of the rats in traversing the beam was classified by using a scoring system: 0 was counted as complete inability to walk on the beam (the animals fell down immediately), 0.5 was scored if the animal was able to traverse half of the beam, 1 point was given for traversing the whole length, 1.5 points were given when stepping with the hindlimbs was partially possible, and 2 points were noted for normal weight support and accurate foot placement. If the scores of all three beams are added, a maximum of six points can be reached[17].

### Hematoxylin and Eeosin (H&E) staining

The H&E staining was performed at the 12^th^ week post injury. Five animals from each group were perfused with 4% paraformaldehyde (Sigma-Aldrich) for histological assessment. The spinal cords were removed from the vertebral columns, fixed with 4% paraformaldehyde for 12 h, and embedded in paraffin (Sigma-Aldrich). Serial sections (thickness of 7 µm) were prepared, deparaffinized with xylol and stained with H&E (Sigma-Aldrich).

### Morphometric assessment

All rats were sacrificed 12 weeks after surgery, and their spinal cords were harvested out and fixed in 4% paraformaldehyde for 12 h. The harvested spinal cord tissues were processed in an automatic processor (Leica TP 1020; Leica, Hamburg, Germany) and embedded in paraffin (Sigma-Aldrich). Serial sections of 7-μm section thickness were made and then deparaffinized with xylol (Sigma-Aldrich). H&E staining was performed on sections of deparaffinized tissue. The mean cavity size was obtained according to Cavalieri’s principle, as cited in Noorafshan *et al*.[18]. The total volume of the spared tissue was obtained by series summation of the spared tissue and cavity volumes. The cavity volume was obtained by using the formula: Vsp=a×d, where “a” is the measured area, and “d” is the intersection distance.

### Immunohistochemistry and BrdU labeling

Three animals from each group were used to evaluate the fate of the cell transplants. The animals were perfused with 4% paraformaldehyde, and the spinal cords were removed from the vertebral column and then transferred to 30% sucrose (Sigma-Aldrich) solution until equilibration. Afterwards, the spinal cord segments were sectioned (8 μm in thickness) and stored at -20°C in a cryoprotectant buffer containing 25% ethylene, 25% glycerin, and 0.05 M phosphate buffer (all from Sigma-Aldrich). To immunostain the sections for mouse anti-BrdU antibody (B8434 Sigma, Sigma-Aldrich), the sections were treated with 50% formamide and 2× saline-sodium citrate buffer (both from Sigma-Aldrich), incubated in 2N HCl (Sigma-Aldrich), washed with 2× saline-sodium citrate buffer, rinsed in Tris (Sigma-Aldrich) buffer saline/0.1% Triton X-100 (Sigma-Aldrich), incubated with the mouse anti-BrdU antibody (Sigma-Aldrich), incubated with goat-anti mouse FITC-conjugated secondary antibody (Sigma-Aldrich), mounted with antifade medium (DakoCytomation, USA), and examined under a fluorescent microscope (Olympus, Japan). Cryosections were attached to silane-coated slides, and the non-specific protein binding sites were blocked with the normal horse serum (Sigma-Aldrich) after washing with PBS. The sections were incubated with mouse monoclonal anti-MBP (Abcam, USA) and diluted to 1:100 in the primary reaction overnight. This process was followed by a similar washing with PBS and 1-h incubation with goat anti-mouse FITC-conjugated secondary antibody (ab6785, Abcam) at a 1:100 dilution in the second reaction. The tissue sections were washed with PBS, and the nuclei were counterstained using ethidium bromide. Then the labeled cells were observed under a fluorescent microscope (Olympus, Japan).

### Luxol fast blue (LFB) staining

The deparaffinized spinal cord sections were incubated in LFB solution (ScyTek Laboratories, USA) at 56°C overnight. After washing with 95% ethanol, the sections were sequentially incubated in lithium carbonate (Sigma-Aldrich) solution and 70% ethanol, each for 30 seconds. The sections were counterstained with cresyl violet (Sigma-Aldrich) solution for 40 seconds and then incubated in 95% ethanol at room temperature for 5 min, dehydrated in 100% alcohol for 5 min, treated with xylene for 5 min and mounted using a resinous medium. Image J software, version 1.46r, was used to assess the myelination quantification.

### Statistical analysis

The statistical analysis was performed using SPSS software, version 16.0. The significance level in BBB scores was analyzed using the analysis of variances (ANOVA). Also, for other statistical comparisons of multiple means in the groups, one-way ANOVA and Tukey’s post hoc test were used. Data in the histograms are presented as mean±SEM. *P* values <0.05 were considered to be statistically significant.

## RESULTS

### Oligodendrocyte-like cell generation and immuno-staining

**Fig. 1:**
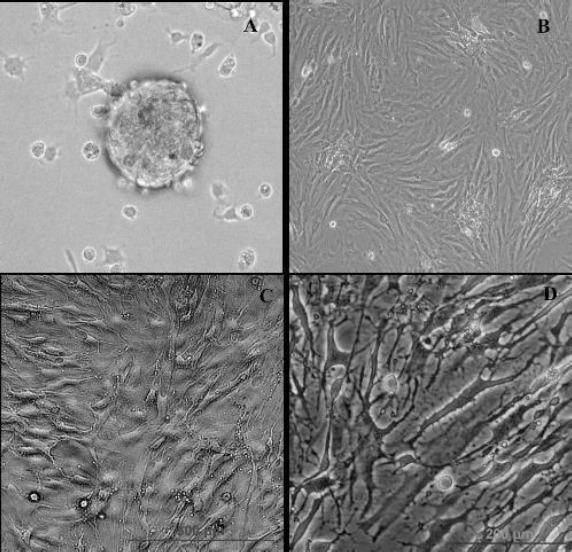
Oligodendrocyte-like cells (OLCs) preparation. After two days of incubation, the neurosphere-like clusters were seen (A). For neural stem cell (NSC) generation, the neurospheres were harvested seven days after plating and seeded on the poly-L-lysine-coated coverslips in six-well culture plates (B). Afterward, to differentiate NSCs into OLCs, NSCs were induced with heregulin and triiodothyronine for four days (C and D). Scale bar=875 µm.

**Fig. 2:**
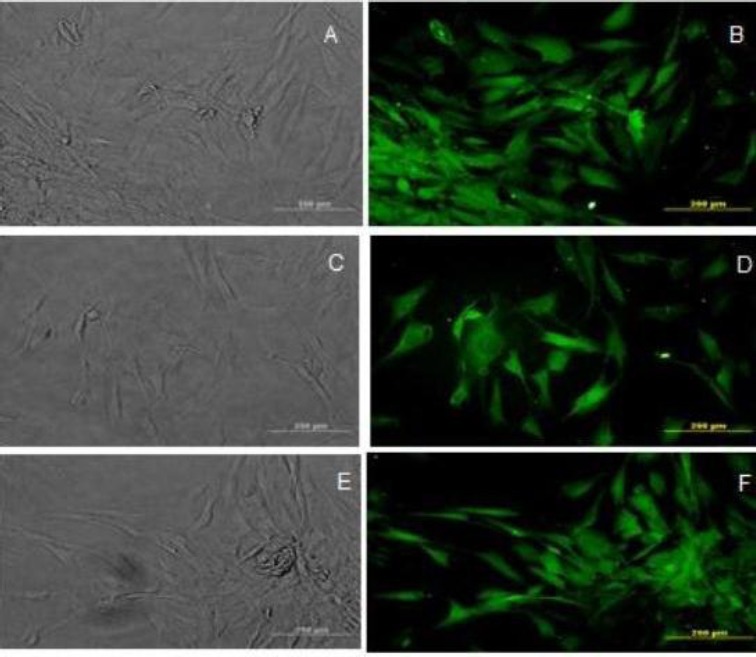
Immunocytochemistry analysis of the transdifferentiated bone marrow stromal cells into oligodendrocyte-like cells. Phase contrast and immunostained cells with anti-O1(A and B), anti-O4 (C and D), and anti-myelin basic protein antibodies (E and F). Scale bar=875 µm.

Figures 1 and 2 represent the phase contrast and immunostaining of induced BMSCs to express O4, O1, and MBP, respectively; the transdifferentiated cells were immunoreactive to these markers.

### qRT-PCR results

**Fig. 3:**
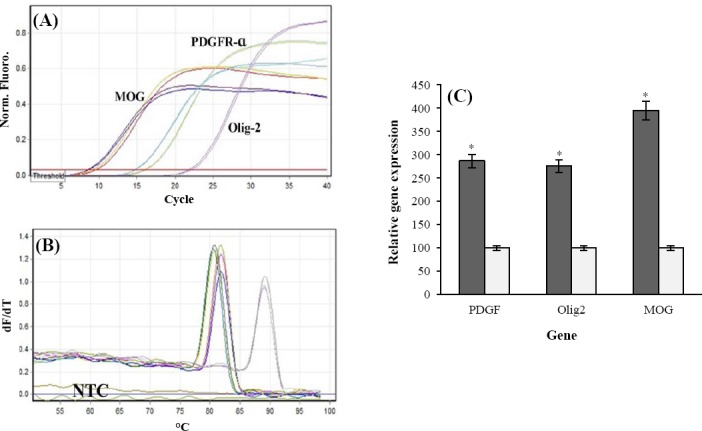
Quantitative RT-PCR. (A) Amplification plots in real time-PCR analyses for all of the amplicons; (B) melt-curves in real-time RT-PCR analyses for all of the amplicons; (C) the relative quantitative expression of PDGFRα, OLIG2, and MOG in OLCs (light gray) using real-time RT-PCR as compared to rat newborn’s spinal cord (deep gray). The expression differences between the two groups were statistically significant. *P*<0.05 (mean±SEM) is shown by a star.

Pfaffl formula[14] was used for calculating the relative qRT-PCR. The results indicated that the OLCs transcribed MOG, OLIG2, and PDGFRα mRNAs nearly 2.7, 2.7, and 3.9fold higher than the rat neonate brain, as compared to control cells, respectively (Fig. 3).

### Behavioral assessment

**Fig. 4:**
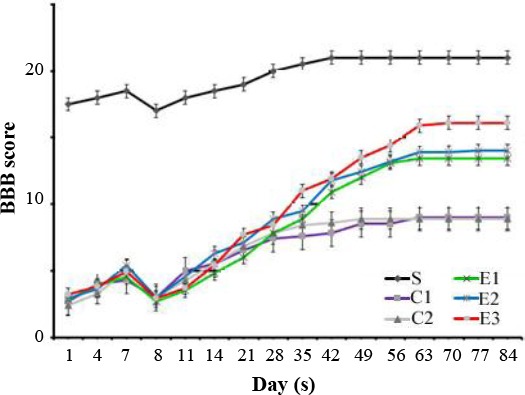
The BBB scores were increased in the treatment period; however, the scores of the OLCs-treated group were significantly higher than those of other experimental groups over significantly higher than those of other experimental groups over time (*P*<0.05). Also, no significant difference was observed in BBB score of the treatment groups on the 8^th^ day.

BBB locomotor rating scores were evaluated for a 12-week period following the contusion model induction in the experimental groups. The mean BBB scores of all groups revealed that the scores were gradually increased from day eight post injury induction. Moreover, on the day eight of cell transplantation, the scores were declined sharply in all experimental groups. The hind limb function was recovered significantly in the COLCs and at low levels in the CNSCs groups in the 12^th^ week post lesion induction in comparison with that of the control groups. The differences among groups were observed to be more significant over time, especially on the 12^th^ week (Fig. 4). The statistical analyses from the first day of the second week until the end of the 12^th^ week showed that the BBB scores were gradually increased. However, the scores of the COLCs experimental group were significantly higher than those of the other groups (S, C, CN, and CB, CNSCs groups) over time (*P*<0.05).

### Narrow beam test

**Fig. 5:**
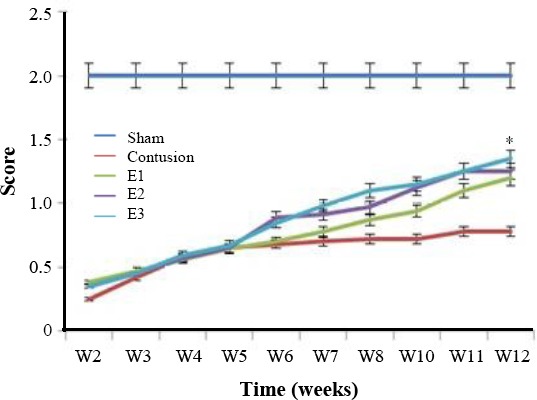
Narrow beam test. Sensorimotor test represented higher function recovery in the implanted group as compared to the control group at week 10 (*P<0.05)

Rats had no errors in their food placement, while they were crossing the beam, before the injury. The rats had poor performance on narrow beam after the injury induction (Fig. 5). The rats’ performances improved gradually, which was evident in animals crossing the beam six weeks after the surgery.

### H&E staining of spinal cord cavities

**Fig. 6:**
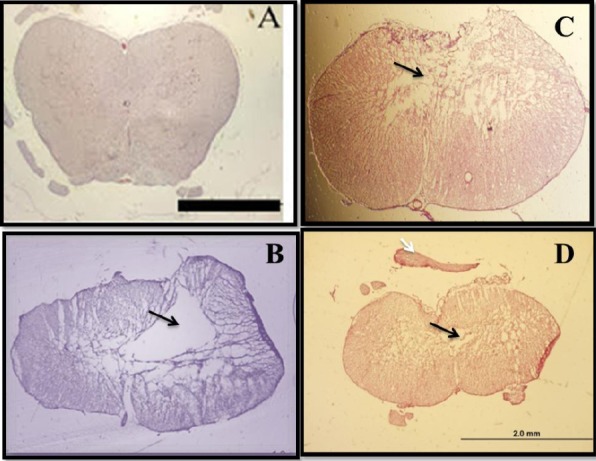
Hematoxylin and eosin (H&E) staining of spinal cord cavities at the lesion site of contusion model. The H&E staining of sections at the lesion site at 12^th^ week post contusion demonstrated that the cell density in the cavity center was much higher in the treated spinal cord, and the cavity size was much smaller as compared to those of the other experimental groups. The H&E staining are as follows: (A) laminectomy only or sham (S); (B) contusion only; (C) contusion+BMSCs transplantation; (D) contusion+neurosphere-derived oligodendrocytes-like cells transplantation. The arrows show the cavity center (scale bar= 1.5 mm).

Morphological changes were explored at the lesion site in all experimental groups. The H&E staining of the spinal cord sections during week 12 showed that the contusion cavity contained transplanted cells in the CB, CNSCs, and COLCs experimental groups. However, the latter group had more resident cells relative to other groups. Also, the cell density around the damaged area in the cell transplanted spinal cord was much higher, and the size of their cavity was smaller as compared to that of the other groups (Fig. 6).

### Cavitation analyses

**Fig. 7:**
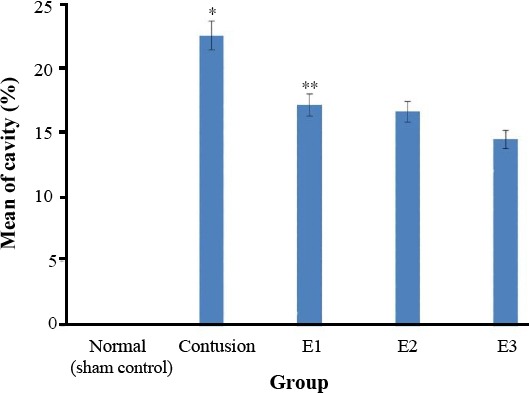
Mean cavitation percentages in all experimental groups. The mean cavitation percentage in the laminectomy only or sham (S), contusion only, contusion+BMSCs transplantation (E1), contusion+NSCs transplantation (E2), and contusion +neurosphere-derived oligodendrocytes-like cells trans-plantation (E3). ^*^Significant contusion group by other groups. ^**^Significant E1 by other groups except E2. The cavity size was decreased significantly in the E3 group.

The histological sections obtained during 12 weeks after the injury were used to evaluate the mean cavity sizes. Image J software, version 1.46r, was used to assess the mean cavity size of the 4900-µm long injured spinal cord. The difference between two groups of E1 and contusion was statistically significant. A significant difference was also observed between weeks 3 and 4 post-injury groups. Data analysis is shown in Figure 7.

### BrdU immunohistochemistry

**Fig. 8:**
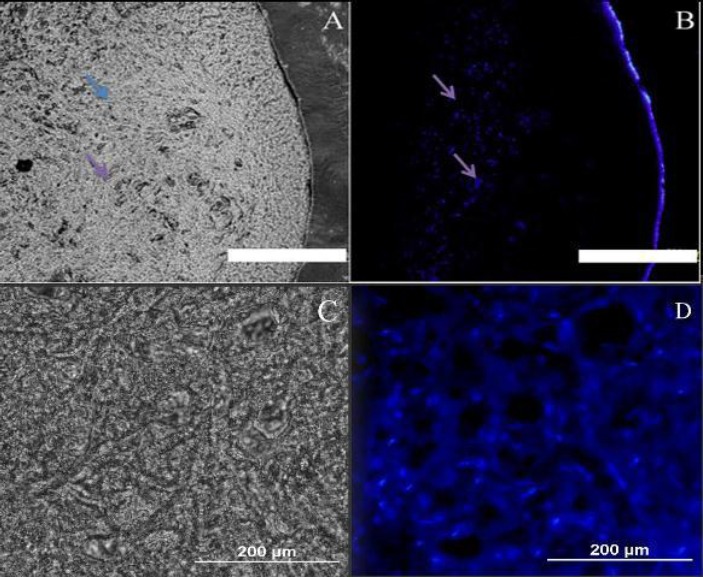
Immunohistochemistry of the transplanted cells. A and B represent the phase contrast and fluorescent labeling using BrdU, respectively. The transplanted cells are scattered throughout both the white and gray matters. They are especially prominent around the cavity center. The BrdU-labeled cells were not observed in the S, C, and CN throughout the gray and the white matters as expected (experimental groups without any cell transplantations). Many of the transplanted cells had aggressively migrated toward the glial scar from the region rostral of the lesion site, which was significantly different from that in the control group (Scale bar=625 µm). Arrows show the transplanted cells, which could survive in the new niche.

For exploring the distribution of the transplanted cells, the immunohistochemical analyses revealed that the BMSCs and the NSCs were scattered throughout the spinal cord sections. They were especially prominent around the cavity center in the CB and CNSCs groups (Fig. 8). The data showed that the transplanted cells survived at the lesion site at least 11 weeks after cell transplantation in all experimental groups.

### Luxol fast blue staining

**Fig. 9:**
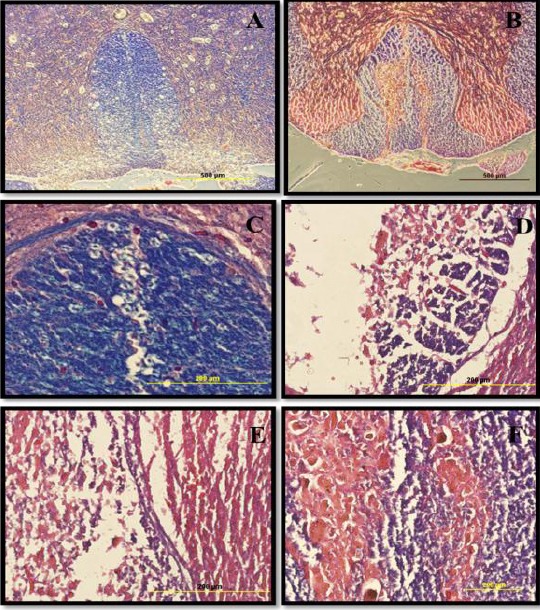
Luxol fast blue (LFB) staining. In order to investigate the effect of oligodendrocyte-like cells (OLCs) on myelin preservation, the extent of residual myelination, stained with LFB, was examined at the injury site 11 weeks post contusive spinal cord injury. (A) laminectomy only, (B) contusion+neurosphere-derived OLCs transplantation (E3), (C) laminectomy only, (D) contusion only, (E) contusion+BMSCs transplantation (E1), and (F) contusion+neurosphere-derived OLCs transplantation (E3) (C, D, E, and F, scale bars=875 and 200 µm). The amount of residual myelin was significantly increased in the OLCs-treated group (*P*<0.01).

In order to investigate the effect of the OLCs on myelin preservation, the contused spinal cords in all experimental groups were stained using LFB, examined at the lesion site 12 weeks post contusive SCI induction. As shown in Figure 9, the amount of residual myelin, stained by LFB, was significantly increased in the OLCs-treated group, and the cavity was re-filled by the transplanted OLCs, which could make myelin substance (*P*<0.01).

### MBP immunostaining

**Fig. 10:**
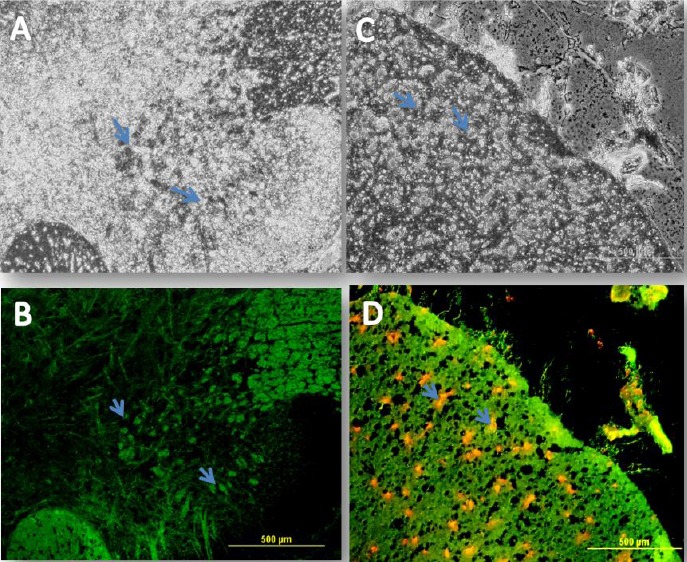
Immunohistochemical staining of myelin. Phase contrast (A and C) and fluorescent labeling of myelin basic protein (MBP) (B and D). In order to further determine whether the transplanted OLCs into the spinal cord injury could regenerate a central nervous system myelin sheath rather than a retrograde dedifferentiation into bone marrow stromal cells in the repaired spinal cord injury site, immunofluorescence staining was performed using an anti-myelin MBP antibody so as to detect this protein in the re-filled areas by the transplanted cells. As shown in the Figure, the transplanted oligodendrocyte-like cells successfully expressed this protein marker in the cavity site. The cell nuclei were counterstained with ethidium bromide. Arrows show the BMP expression and nuclei staining simultaneously (scale bar=500 µm).

The injured spinal cords with OLCs transplantations could regenerate the myelin sheath in the central nervous system, which was investigated using MBP immunofluorescence by anti-myelin MBP antibody so as to detect the presence of this protein in the re-filled areas by transplanted OLCs (Fig. 10). As shown in this Figure, the transplanted OLCs successfully expressed this protein marker in the cavity. The cell nuclei were counterstained using ethidium bromide.

## DISCUSSION

In the present study, the neurosphere-derived OLCs were transplanted into SCI of a rat model, and the results showed that these cells were a feasible source for cell therapy of contused rats. Rat models receiving neurosphere-derived OLCs had a better recovery than the untreated rats, according to BBB test scores. Finding a cure for CNS injuries remains a challenge in the field of regenerative medicine. It is well accepted One of the pathological outcomes of acute and chronic CNS trauma is axonal demyelination. Even though the re-myelination is mediated by proliferating and migrating endogenous stem cells into the demyelinated zone[19], it is insufficient and far from bringing recovery. Indeed, this re-myelination never reaches completion. Oligodendrocytes are particularly that spinal cord trauma results in an immense cellular damage at and around the lesion site. The primary physical assault causes rupturing of vessels, subsequently hemorrhage and eventually necrosis of the resident neuronal and glial cells[20].

vulnerable to oxidative stress and excitotoxicity due to their high metabolic activity, high levels of intracellular iron, and low concentration of glutathione[21]. An approach to counterbalance this situation, which is relied on the treatment, is re-filling the oligodendrocyte pool with BMSCs-derived neurospheres transdifferentiated into OLCs after injury. The cavity resulted from spinal trauma was reduced in size in the experimental groups that received the transplanted cells after 12 weeks of cell transplantation. Another aspect of traumatic CNS cell therapy is transplanted cell survival[22].

In the current study, the implanted BMSCs and NSCs survived for at least 12 weeks at the niche of lesion site that was confirmed with BrdU labeling results. As the DNA synthesized, the BrdU (a synthetic analog of thymidine) incorporated into the daughter strands. This integrated analog of thymidine into the DNA could be detected in the tissue. LFB staining showed that the amount of myelin was significantly increased in the OLCs-treated group compared to other experimental groups. Moreover, LFB staining indicated that transplanted OLCs could adapt to the new niche and survive.

To further determine whether the transplanted OLCs can regenerate myelin sheath of the central nervous system rather than a retrograde dedifferentiation to BMSCs in the repaired SCI site, MBP immunostaining was performed. Interestingly, in the new niche, the OLCs expressed MBP marker. The evidence implies that transplantation of neurosphere-derived OLCs would be an effective therapeutic strategy that can swing the balance toward the survival of oligodendrocytes over cell death. However, it is well accepted that in SCI many things go awry, and thus it is very unlikely that a treatment based on a single intervention, such as one drug or one type of cell implantation, would be able to counteract all or most aspects of the damage. Accordingly, more experimental studies are needed for combining different treatments so that to obtain the best combination for injury recovery. Additional alternative approaches that can be combined with cell therapy include delivery of neurotrophic factors and providing scaffolds for regeneration[23-26]. Delivery of different types of cells and also effective drugs is aimed to provide trophic support, help re-myelinate denuded axons, serve as a conduit for nerve growth and replace or substitute for the lost neurons[24,26]. Combining growth factor treatment with genetic manipulation of stem cells with the desired differentiated/ transdifferentiated cells would be an ideal option for treatment of SCI[25, 27].

Collectively, many factors exert their detrimental effects on the oligodendrocyte cell viability, and one of the conceptions to alleviate oligodendrocyte loss during spinal cord trauma occurrence is administration of OLCs to prevent the ensuing axonal demyelination. In the current study, the BMSCs were trans-differentiated into neurospheres and then into OLCs, and the functional recovery after cell transplantation was explored and interpreted in comparison with control groups. We have modified the method presented by Kaka *et al*.[27] as they did not prepare NSCs from neurospheres. In the present study, we actually transdiffrentiated the BMSCs into neurosphere clusters and then into NSC. These NSCs were then differentiated into oligodendrocytes cells using triiodothyronine. In comparison with the aforementioned work, the efficiency of trans-diffrentation in our study was significantly higher. The BBB scores also were much higher as compared to BMSCs- and non-neurosphere-derived oligodendro-cytes. Our findings show that OLC therapy could act as a potential approach for treating spinal cord traumatic injuries.
